# Do Exercise-Based Prevention Programs Reduce Injury in Endurance Runners? A Systematic Review and Meta-Analysis

**DOI:** 10.1007/s40279-024-01993-7

**Published:** 2024-01-23

**Authors:** Han Wu, Katherine Brooke-Wavell, Daniel T. P. Fong, Max R. Paquette, Richard C. Blagrove

**Affiliations:** 1 National Centre for Sport and Exercise Medicine, School of Sport, Exercise and Health Sciences, Loughborough University, Loughborough, UK; 2 College of Health Sciences, University of Memphis, Memphis, TN USA

## Abstract

**Background:**

Endurance running is a popular sport and recreational activity yet is associated with a high prevalence of injury. Running related injuries (RRIs) are a leading cause of drop-out and represent a substantial financial burden to runners and healthcare services. There is clear evidence for the use of exercise-based injury prevention programs in games-based and youth sport settings, yet the research investigating the use of exercise to reduce injury risk in endurance runners has not been adequately reviewed recently.

**Objectives:**

The aim of this review and meta-analysis was to systematically summarize the current research that has investigated the effect of exercise-based prevention programs and their state of supervision on the risk of RRIs in endurance runners.

**Methods:**

Three databases were searched for relevant studies. Selection and review were completed by two independent reviewers using the following inclusion criteria: (1) study population used endurance running training for health, occupational, or performance outcome(s); (2) participants performed running as their main form of exercise (> 50% of their total training time); (3) study was a randomized controlled trial; (4) a non-running-based exercise intervention was used; (5) a running-only or placebo exercise control group was included; (6) injury rate or incidence was reported; (7) injuries were recorded prospectively alongside the exercise training. Two meta-analyses were conducted using random-effects models, one based on log risk ratio and one based on log incidence rate ratio. The Cochrane Risk of Bias Assessment Tool 2 was used to evaluate the quality of studies and the Grading of Recommendations Assessment, Development and Evaluations approach was employed to grade the certainty of evidence.

**Results:**

A total of nine articles containing 1904 participants were included in analysis. Overall pooled results showed no significant differences between intervention and control groups in injury risk (*z* = − 1.60; *p* = 0.110) and injury rate (*z* = − 0.98; *p* = 0.329), while a post hoc analysis evaluating supervised interventions only showed that injury risk was significantly lower in the intervention group compared to the control group (*z* = − 3.75, *p* < 0.001). Risk of bias assessment revealed that seven studies included in the analysis were of low quality.

**Conclusions:**

Exercise-based interventions do not appear to reduce the risk and rate of running-related injuries. Supervision may be essential for exercise-based intervention programs to reduce risk of RRIs, possibly due to increased compliance. Studies with more robust designs that include supervised exercise interventions should be prioritized in the future.

**Trial Registry:**

Clinical Trial Registration: PROSPERO CRD42021211274.

**Supplementary Information:**

The online version contains supplementary material available at 10.1007/s40279-024-01993-7.

## Key Points

**Table:** 

Injury prevalence is high in endurance runners; however, the research investigating the use of exercise to reduce injury risk specifically in endurance runners has not been reviewed adequately.
Pooled data showed that exercise-based injury prevention programs provide no reduction in injury risk or injury rate compared to running only.
Studies that used an element of supervision during interventions tended to have greater compliance with the exercise programs and showed significantly lower injury risk compared to control groups.
Most studies in this area are of low quality, indicating that future research should use more robust study designs with supervised exercise interventions.

## Introduction

Endurance running is a popular physical activity associated with a myriad of health benefits such as reduced risk of non-communicable diseases and improved mental well-being [1, 2]. Participation in community endurance running events and initiatives has increased substantially in recent years; for example *parkrun* attracts 330,000 people across 2200 events worldwide every weekend [3] and over six million runs were completed via the UK National Health Service ‘*Couch to 5k*’ application in 2022 [4]. In addition to public participation, endurance running is also an integral component of military training [5], is a popular sport, and constitutes a major component of training for athletes in other sports such as triathlon and duathlon.

Endurance running is associated with a high incidence of running-related injuries (RRIs) [6, 7]. A recent systematic review encompassing over 10,000 runners noted that both injury incidence and prevalence exceed 40% [7]. In the military, running volume has been shown to be a leading cause of injury, contributing to 13.5% of all injuries and 34.6% of preventable injuries [8]. Although elite runners tend to have fewer RRIs compared to novice runners, injury incidence is still high [9]. An observational study containing 4621 runners found injury incidence to be 8.78 for novice runners and 4.24 for experienced runners per 1000 h of running [8]. RRIs are mainly lower limb overuse injuries, such as patellofemoral pain, medial tibial stress syndrome, and Achilles tendinopathy [7]. Overuse injuries generally require a long recovery time and are often the reason for stalled progress, pre-race drop-out [10], and runners quitting the sport [11]. Injuries represent a substantial financial burden to runners, health services and employers, which may be reduced if effective injury prevention strategies were available and utilized [12].

In the presence of a high incidence of RRIs, preventative recommendations have been put forward by academics and medical professionals despite an absence of compelling evidence to support these suggestions [13–15]. Incorporation of strength training activities (e.g., resistance and plyometric training) and other therapeutic exercise interventions (e.g., stretching, proprioception exercises, core stability exercises) into a running program are common recommendations [13–15]. Indeed, it has been noted that a high proportion of endurance runners engage in strength and conditioning (S&C) activities due to the belief it lowers the risk of sustaining a RRI [16, 17]. Notwithstanding the popular use of S&C activities, previous reviews showed no clear evidence for stretching [18, 19] and conditioning exercises to lower RRI risk [19, 20]. However, the definition of ‘runners’ has been poorly defined in the two existing reviews investigating the effects of S&C and therapeutic exercise on RRIs [19, 20]. Specifically, these two reviews included studies in military populations, which assumes injuries sustained during basic military training activities are due to running activities and are the same as endurance RRIs. Although running often constitutes a large part of military training, it may not be a major component for all branches of the military used across different studies. For example, in the three overlapping military studies included in these two reviews, endurance running only constituted 21% of the physical training routine [5, 21, 22].

Prospective studies have identified that muscular weakness predisposes runners to a higher incidence of patellofemoral pain [23], medial tibial stress syndrome [24], and Achilles tendinopathy [25]. From this standpoint, engaging in activities such as resistance training that improve muscular strength may therefore reduce the risk of RRI. However, a review that systematically evaluates whether these relationships are causal in nature has not been conducted recently for endurance runners. In the broader literature, the protective effect of ‘neuromuscular training’ programs, containing combinations of strength, agility, balance, core stability, plyometric and bodyweight exercises, on the incidence of sport injuries in games players [26, 27] and youth athletes [28] is well established. Specifically, multicomponent exercise programs have been shown to reduce the risk of sustaining an overuse injury by almost half, whereas stretching alone provided no protective effect [29]. A recent review also found that strength training provided a dose-dependent sports injury risk reduction [30]. Despite these promising findings, these reviews did not include any studies that used endurance runners, who experience high volumes of repetitive cyclical loading of musculoskeletal structures, and therefore it is currently unknown whether the results are applicable to this population.

A review of the published literature that systematically evaluates whether exercise interventions reduce the risk of RRIs, specifically in endurance runners, is warranted. This information would be useful for sports medicine practitioners, coaches and runners to make more informed decisions when selecting injury prevention strategies. Furthermore, a detailed examination of the protocols used in previous studies will help identify limitations in study design and implementation, thereby directing further research in this area. Consequently, this systematic review and meta-analysis aims to provide an update on the current evidence surrounding the effect of exercise programs on the risk of RRIs.

## Methods

This study was registered a priori on PROSPERO (CRD42021211274) and the updated PRISMA statement [31] and PERSiST guidance [32] were used as a basis for the procedures described herein.

### Inclusion and Exclusion Criteria

For a study to be eligible at the systematic review stage, the following inclusion criteria were met:

- Study population used endurance running training for health, occupational (e.g., military preparation), or performance (5 km—ultra-endurance events) outcome(s). When endurance runners composed a subset of the study population (e.g., track-and-field athletes), the study was initially included and the corresponding author contacted to obtain endurance runners’ data.
- Participants performed running as their main form of training, defined as running accounting for > 50% of their total training time during the study period.
- Study was a randomized controlled trial.
- A non-running-based exercise intervention was used.
- A running-only or placebo exercise control group was included.
- General or specific injury rate or incidence was reported as an outcome measure.
- Injuries were recorded prospectively alongside the exercise training.
- Published in full in a peer-reviewed journal (excluding pre-prints).

Studies were excluded if any of the following applied:

- Participants were non-runners or endurance running formed ≤ 50% of the overall training program. Restrictions were not placed upon experience/training status. Where there was doubt over the volume of endurance running relative to the overall exercise training programme, corresponding authors were contacted.
- The running training and/or intervention was not clearly reported.
- Participants were injured at baseline (i.e., a rehabilitation intervention was used) or reported to be in poor physical health or symptomatic.
- Pharmaceutical or other non-exercise prevention strategy (e.g., orthotics, independent massage sessions, nutrition) was used alongside an exercise intervention.

### Systematic Search, Study Selection and Data Extraction

A search was conducted in PubMed, Web of Science and SPORTDiscus on 15 January 2023, with no publication date or language restrictions. The search was divided into blocks of keywords and associated synonyms relating to ‘prevention’, ‘injuries’, ‘non-running exercise’, ‘running’, and the study design. Blocks were separated by the operator ‘AND’ and contained the operators ‘OR’ and ‘*’ (see Electronic Supplementary Material (ESM) Appendix S1 for full operational search strings). Title/abstract screening and full-text screening were conducted by two independent reviewers (HW and RCB) using the web-based systematic review tool Covidence (Veritas Health Innovation). Citations were screened in the reports that were assessed for eligibility. No automated tools were used in the identification and screening process. Data extraction and the risk of bias assessment were conducted by the same two reviewers using a modified version of the data extraction form recommended by the Cochrane Handbook for Systematic Reviews of Interventions [33] and the risk of bias 2 Excel Macro Form (Beta version 7), respectively. Inter-rater reliability (IRR) percentage agreement and a kappa coefficient statistic (*k*) were calculated for each stage of the process. Discrepancies regarding screening, extraction, and risk of bias assessment were resolved via discussion between the two reviewers.

### Meta-Analysis

Two meta-analyses were conducted using random-effects models: one based on injury risk (injured divided by all athletes during the study period), for which the log risk ratio was used as the outcome measure; and a second based on injury rate (number of recorded injuries per 1000 training hours), for which the log incidence rate ratio was used as the outcome measure [34]. Corresponding authors of studies that lacked either injury rate or injuries per 1000 h were contacted for further data, and studies were excluded from meta-analysis if relevant data were not available. For studies that had both intention-to-treat and subgroup analysis data available, intention-to-treat data were used preferentially. For studies that had multiple intervention groups, participants from all intervention groups were pooled for analysis. Data analysis and presentation were conducted using metafor (version 3.4.0), a meta-analysis tool package in R [35]. The code used for the meta-analysis in R is provided in ESM Appendix 2. Cochrane’s *Q*-test, *τ*^2^, and *I*^2^ statistics were calculated for heterogeneity. A 95% prediction interval for the true outcome was calculated if heterogeneity was present, which was defined as *τ*^2^ > 0 [36]. Potential outliers and single studies that may be too influential were identified using the studentized residuals and Cook’s distances [35]. A threshold for studentized residual was set to be $100\times \left(1-0.05/\left(2\times k\right)\right)$th percentile of a standard normal distribution, and a threshold for Cook’s distance was set to be median plus six times the interquartile range. A funnel plot was produced for the injury risk meta-analysis, and asymmetry was checked using Spearman’s rank correlation coefficient [37] and linear regression [38].

An additional post hoc meta-analysis was performed on studies that utilized supervision during the intervention. The outcome measure was set to be injury risk (the number of injured participants divided by the number of participants in the study) and all procedures were in line with the meta-analysis on injury risk described above, except a funnel plot was not produced [33].

### Deviations from Pre-registered Protocol

The protocol described herein deviated from the PROSPERO-registered protocol in the following ways:

- Originally, it was proposed that risk of bias would be evaluated using both the Cochrane tool and the PEDro scale, but to avoid confusion, only the Cochrane tool was employed. An additional certainty of evidence assessment using the Grading of Recommendations, Assessment, Development and Evaluations (GRADE) approach was added based on reviewer suggestions.
- It was not possible to calculate accurate Hedges’ g effect sizes values for most of the included studies, and therefore these are not reported in Sect. 3.
- Sub-group analysis for training modality was planned in the pre-registered protocol; however the majority of studies used mixed-mode exercise interventions, and sub-group analysis was therefore redundant. Sub-group analysis for training supervision was added as clear differences were noted in the way interventions were administered in this respect.

### Grading of Recommendations, Assessment, Development and Evaluations

To rate the certainty of the evidence provided by this review, the Cochrane GRADE approach was used [39]. Assessments were conducted on injury risk and injury rate which were the two outcomes analyzed in the meta-analyses. A GRADE evidence profile and a summary of findings table were created in accordance with GRADE Handbook instructions [39]. Each of the GRADE criteria were judged by the following methods: (1) risk of bias, by inspecting the risk of bias 2 assessment results and evaluating whether the ‘high risk’ sections lowered the confidence in the estimate of effect; (2) inconsistency, by inspecting point estimates and confidence interval overlaps among studies; (3) indirectness, by inspecting whether the study population and intervention were directly applicable to our target topic; (4) imprecision, by considering the optimal information size and the overlap between the confidence interval and no effect; (5) publication bias, by inspecting the funnel plots for inexplicable asymmetries [39].

## Results

### Search Results

**Fig. 1:**
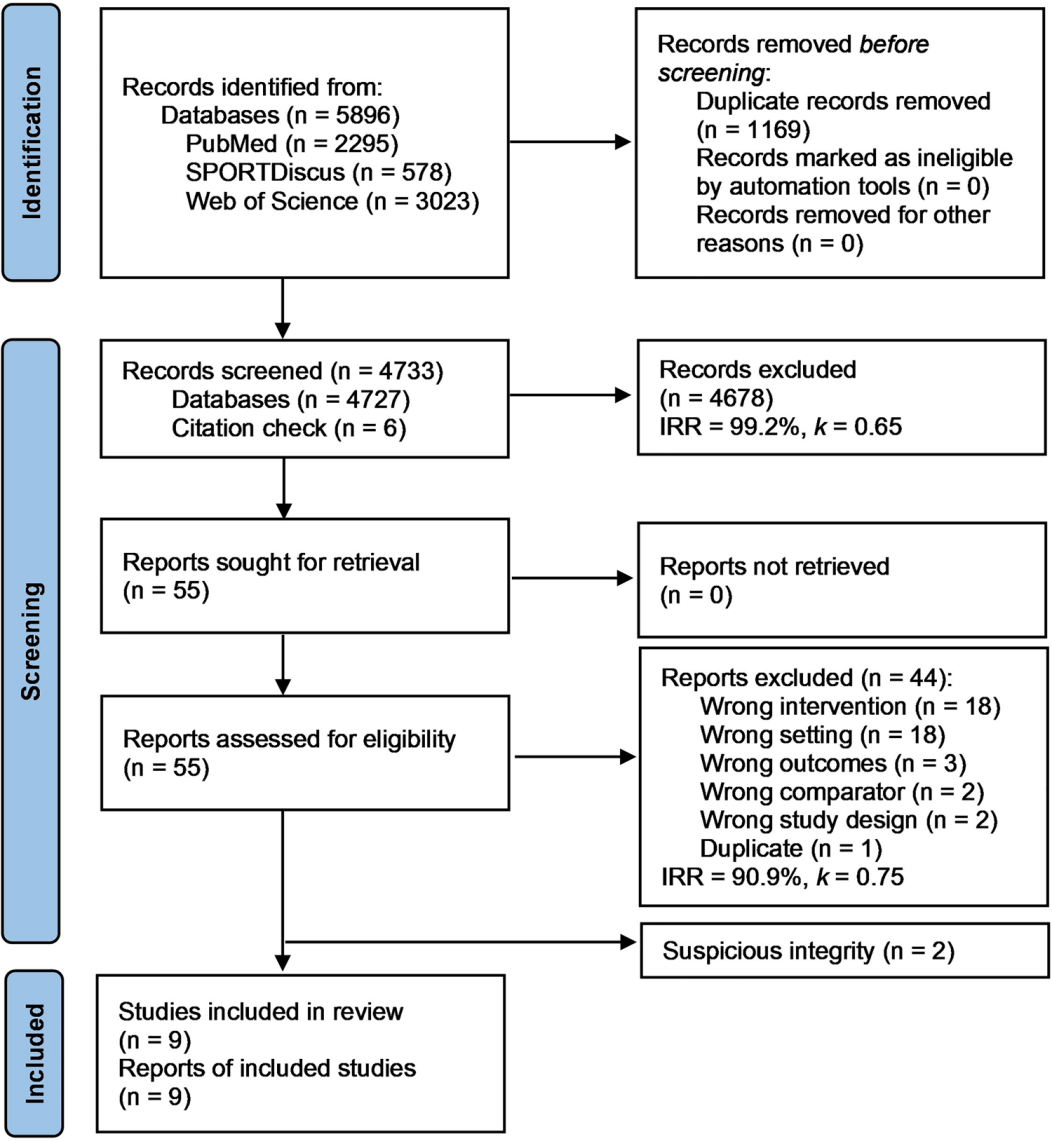
Systematic review search, screening, and selection process

Figure 1 shows a flowchart summarizing the screening process. The initial search yielded 5896 results with 1169 duplicates. Citation checking yielded an additional six papers leaving 4733 for title and abstract screening. The eligibility screening stage excluded 4678 articles (IRR = 99.2%, *k* = 0.65), leaving 55 articles that were taken forward for full screening. Within these 55 studies, authors of eight studies involving military participants were contacted due to an unspecified volume of running training completed relative to total training volume. Four responses were received, each confirming that physical training in the study involved < 50% endurance running in both groups. All eight studies were therefore excluded from further analysis. Another 36 articles were also excluded at this point, meaning the number of articles excluded after full-text screening was 44 (IRR = 90.9%, *k* = 0.75; ESM Tables S1–S2), leaving 11 papers for data extraction.

During data extraction, it was identified that two studies [40, 41] shared inexplicably similar data that could not be reconciled. These two studies were conducted by the same first author, and participants within the studies shared the same baseline data for age, height, body mass, body mass index, running experience, and several biomechanics parameters measured during treadmill running. However, the studies had different sample sizes and used interventions of different durations (6 vs. 8 weeks) and exercises. The studies reported identical injury occurrences during the prospective 1-year follow-up period, which meant including both in the meta-analysis would produce a duplicate data point. The corresponding author of the two articles was contacted twice; however, no response was received, and these two papers were therefore also excluded, leaving a total of nine studies.

### Study Characteristics

Table 1 provides a summary of the study characteristics included in the review and Table 2 summarizes the training undertaken by participants. A total of 1904 participants were included in the analysis. Seven studies used a mixed-sex cohort of participants [42–46, 48, 49], one used only males [50] and one used only females [47]. Three investigations used competitive level athletes [44, 45, 47], and six reported using novice or recreational runners [42, 43, 46, 48–50]. Eight studies were performed on adults [42–46, 48–50] and one used adolescents [47].

**Table 1:** Study designs and participant characteristics

Study	Study origin	Intervention duration	Sex (*n*)	Participant n in analysis	Participants	Training status	Age (years)	Intervention	Control group	Group allocation
Baltich et al. [42]	Canada	6 months	M (26) F (103)	RT: 43 FSST: 43 Stretch (con): 43	Healthy novice recreational	Recreational (< 2 years’ experience)	18–60 (Median RT: 30, FSST: 33, Stretch 31)	RT (ankle focused) vs. FSST	5 min shuffling, skipping, light running + static and dynamic stretching (25 min)	Unstratified randomization
Bredeweg et al. [43]	Netherlands	4 weeks (9-week follow-up)	M (149) F (283)	Precon: 171 Con: 191	Healthy novice recreational	52% with some running experience	18–65 (mean ± SD Precon: 39.0 ± 10.7, control 37.2 ± 10.9)	Precon (walk and hop) before running training period	No exercise (including running) before running training period	Stratified randomization (current sporting activities, previous injury, sex)
Edouard et al. [44]	France	40 weeks	M (490) F (350)	Int: 59 Con: 77	Competitive club level athletes	–	15–40	Core stability, balance, strength exercises, stretching	Running training only	Cluster stratified randomization within Athletics Clubs
Halvarsson and von Rosen [45]	Sweden	14 weeks (pre-season wk 1–4, competitive season wk 5–14)	M (32) F (30)	Int: 30 Con: 32	Elite orienteerers	Int: 5.8 ± 3.3 years Con: 6.6 ± 3.6 years	18–40 (Int: 24.1 ± 3.5, Con: 24.2 ± 3.8)	NM control (balance and jumping exercise)	Running training only	Stratified randomization (sex)
Lundstrom et al. [46]	USA	12 weeks	M (15) F (19)	Core: 12 Plyo: 11 Control: 11	Healthy recreational adults	Recreational	Core: 20.2 ± 1.4 Plyo: 21.0 ± 1.0 Control: 21.0 ± 1.1	Core (exercises for trunk/hips); plyo (jumping and sprinting)	Running training only	Block stratified randomization (sex)
Mendez-Rebolledo et al. [47]	Chile	6 weeks	F (16), sprinters excluded	Con: 8 Int: 8	Adolescent female middle- and long-distance runners	Regional or national level (> 1 year experience)	11–18 Con: 15.9 NM: 16.9	NM training (strength, agility, balance, core, plyometrics, bodyweight exercise)	Running training only	Random sampling (1:1 ratio)
Taddei et al. [48]	Brazil	12 months	M (61) F (57)	Con: 61 Int: 57	Healthy recreational long-distance runners	Con: 6.9 years, Int: 5.4 years (20–100 km per week)	18–55 Con: 41.3 ± 6.8 Int: 40.5 ± 7.9	Foot and ankle conditioning	Static stretching	Block randomization
Toresdahl et al. [49]	USA	12 weeks	M (220) F (500)	Con: 368 Str: 352	Novice marathon runners	Goal marathon time 4 h 34.0 min ± 40.8 min	> 18 Con: 36.3 ± 9.8 Str: 35.4 ± 9.1	Core, hip abductor, quadriceps strength exercises	Running training only	Block stratified randomization (sex)
Van Mechelen et al. [50]	Netherlands	16 weeks	M (327)	Con: 168 Int: 159	Recreational runners	Running > 10 km per week	< 25 *n* = 129; 25–32 *n* = 155; > 32 *n* = 137	Running drills, mobility and stretching	Running training only	Matched stratified randomization (weekly running distance, age, knowledge on prevention of injury)

*RCT* randomized controlled trial, *M* male, *F* female, *RT* resistance training, *FSST* functional sport-specific strength training, *Precon* pre-conditioning intervention, *Con* control group, *SD* standard deviation, *Int* intervention group, *NM* neuromuscular, *Plyo* plyometric training group, *Str* strength training group

**Table 2:** Intervention and running training prescription

Study	Running training	Intervention exercises	Intervention frequency	Intervention volume	Intervention intensity	Supervision/instruction
Baltich et al. [42]	RT: mean 12.5 h FSST: mean 16.3 h Stretch: mean 14 h	RT: mini band exercises FSST: lunge, squat, hops, jumps (flat ground and BOSU) (+ 5 min shuffling, skipping, light running and 5 min static/dynamic stretching)	Both groups: 3–5 × per week for 8 weeks; 2 × per week for 16 weeks	RT: 4 sets × 10 reps; isometric 3 sets × 5 s FSST: Lunge 1 × 10, squat × 10, hop × 5, SL standing 5 × 30 s eyes open, 5 × 30 s eyes closed, jumps × 20	RT: mini band (increasing tension) FSST: bodyweight and BOSU ball	None (home-based); coaching fortnightly and video-based instruction
Bredeweg et al. [43]	Precon: 301.1 ± 184.7 min Con: 329.7 ± 177.1 min NSD	Walk 5 min, hops; 30–60 min walk	Walk/hops 2 × per week 30–60 min walk 1 × per week	Hops 6 × 50–90 reps	Bodyweight	None (initial interview + instruction videos)
Edouard et al. [44]	Sub-group training not defined	Plank (prone/supine/side), single leg balance (unstable), lunges, standing hip abduction, hamstring stretch, hamstring curls, hamstring bridge holds, Nordics, calf stretch, calf raises (single leg)	≥ 2 × per week	~ 15 min total Planks 15–30 s per side (3–12 min total); balance 3 × 15–30 s; lunges/hip abduction 3–6 × 10; stretches 3 × 15 s; hamstring holds 3–6 × 6–10 s; Nordics 1–6 × 5–6; calf raises 3–5 × 8–10	Bodyweight Progressions: standing hip abduction with elastic resistance; balance throw-catch with ball	Unsupervised (paper and video guidance)
Halvarsson and von Rosen [45]	Int 7.2 h/week Con 7.4 h/week NSD	One-leg stance, runners pose, one leg heel raise, and one leg side hop (increase in difficulty fortnightly)	4 × per week	One-leg stance (eyes-closed, soft surface): 2 min/leg Runners pose: 3 sets × 10 reps per leg One-leg heel raise: 3 sets × 15 reps per leg One-leg side hop: 2 sets × 30 reps per leg	Bodyweight Progressions: One-leg stance: add arm/leg movements and knee bends; Runners pose: added heel raise and soft surface Heel raise: on a stair (larger range) without support Hop: 40 cm distance; arms across chest, cervical rotation	Unsupervised (pictures of the exercises and verbal explanation provided)
Lundstrom et al. [46]	Core: 26.2 miles/week Plyo: 28.2 miles/week Con: 27.2 miles/week NSD	Core: crunches, side crunches sit-ups, V-sit-ups, supermans, back extensions, planks, fire hydrant, Swiss Ball adductors, bridging, bird dog Plyo: 50–60 m sprints, hops, bounds, cone jumps, squat jumps, scissor jumps, depth jumps, box jumps	1 × per week	15–20 min per session Core: 1–3 sets × 10–30 reps per exercise or 30–60 s Plyo: 1–3 sets of 8–20 reps per exercise or 2–4 reps per sprinting exercise	Both groups: bodyweight Core: slow-moderate velocity	Yes
Mendez-Rebolledo et al. [47]	3 × per week; Con: 114 min per session Int: 119 min per session. Included aerobic and anaerobic running; weight and circuit training	Dynamic flexibility and agility drills, planks, bridges, superman, squats, crunches, leg raises, calf raises, Y-balance, jumps and hops, RDL, hip thrusts, lunges, Nordic curls,	3 × per week	30 min per session (6–8 exercises per session) Agility drills: 4 × 30 m Jumps and hops: 1 × 10 reps Exercises: 2 × 20 reps or 20 s holds	Bodyweight BOSU for Y-balance; core exercises with therapeutic ball; hip thrust with barbell; calf raises with dumbbells	Yes
Taddei et al. [48]	Con (stretch): 97.7 ± 61.4 km/mo at 6.6 ± 1.4 min/km Int: 82.3 ± 59.5 km/mo at 6.7 ± 1.9 min/km NSD	Int: Massage and manipulation, foot tapping, heel raises, sitting invert/evert, band foot abduction, band toe and ankle flexion, grab-hold ball/pen, toe squeezes, toe abd-/add-uction, short-foot, plantar arch raise, toe grasping Stretch: calf wall, standing quadriceps, standing to toe touch, crossed legs standing to toe touch, seated adductor, pretzel, lying lateral stretch	Int: 4 × per week Con (stretch): 3 × per week	Int: 20–30 min per session (12 exercises) 1–2 sets × 10–30 reps progressing to 3–6 sets or increasing isometric holds or moving from sitting to standing to single leg Con (stretch): 5 min per session; 1 set × 20 s per stretch	Various	Int: Supervised 1 × per week; online exercise descriptions and videos additional 3 × per week with remote supervision Con (stretch): Unsupervised; online descriptions and images
Toresdahl et al. [49]	Con: 3.3 ± 1.1 runs per week Int: 3.4 ± 1.1 runs per week NSD	Bodyweight squat or jump squats, front plank, lunges or jump lunges, side plank (or top leg raise), single leg toe touches (or with jump)	3 × per week	10 min per session Squats: 3 × 20 reps Single leg exercises: 2 × 10 each side Plank positions: 1 × 0–60 s	Bodyweight	Unsupervised (instructional video and handout provided)
Van Mechelen et al. [50]	Con: 344 ± 259 km, 2.6 ± 1.3 per week, 12.4 ± 1.5 km/h; Int: 370 ± 263 km, 2.8 ± 1.3 per week, 12.4 ± 1.7 km/h NSD	Warm-up: running drills, loosening exercises, stretching (hip flexors, hamstrings, calf) Cool-down: inverse of warm-ux p	Warm-up/cool-down with any running sessions Stretching 2 × per day	Running drills: 6 min Loosening exercises: 3 min Stretching: 10 min (3 sets × 10 s each stretch)	Bodyweight	Unsupervised (instructional booklet and one group coaching session)

*RT* resistance training, *FSST* functional sport-specific strength training, *reps* repetitions, *Precon* pre-conditioning intervention, *Con* control group, *NSD* not statistically different, *Int* intervention group, *Plyo* plyometric, *NM* neuromuscular, *RDL* Romanian deadlift

Studies used a variety of exercise modalities as part of interventions. Most studies used a multi-modal activity approach within sessions, including jumping or plyometric exercises [43, 45–47], multi-joint and single-joint strength exercises [42, 44, 47, 49], exercises focused on the core or trunk musculature [44, 46, 47, 49], balance or proprioception exercises [42, 44, 45, 47], stretching or mobility exercises [44, 47, 50], and sprint/running or agility drills [46, 47, 50]. All studies mentioned that exercises were progressed in terms of complexity/difficulty and/or progressive overload was achieved via increases in volume or intensity of the exercises. Of the studies that utilized an element of strength training, bodyweight was used as resistance on most exercises, with elastic bands used to progress intensity in two of the studies [42, 44], and free weights used in two exercises in another study [47]. One study used foot and ankle strengthening exercises including progressive overload with elastic resistance [48].

Within the nine included studies, three studies used interventions that were supervised, meaning that online or in-person supervision was provided at least once per week during the intervention period [46–48]. The other six studies used no supervision, providing instructional materials such as online videos, demonstrative booklets, and/or a single demonstration session [42–45, 49, 50].

Overall, studies with supervision achieved better compliance. Studies calculated compliance in two different ways. The three studies with supervision all reported number of attended sessions in relation to total training sessions and the percentages were ≥ 88% [46–48]. All unsupervised interventions reported the percentage of participants completing more than a specified number of the total sessions or sessions per week; five of these studies reported compliance of 47–72% [43–45, 49, 50], and one reported 86–93% [42].

Intention-to-treat analysis was performed on five articles [42, 45–49]. Within these five studies, three performed additional subgroup analysis for compliant participants [42, 45, 49]. Mendez-Rebolledo and colleagues [47] reported full compliance and thus intention-to-treat analysis was not applicable.

### Risk of Bias Assessment

**Fig. 2:**
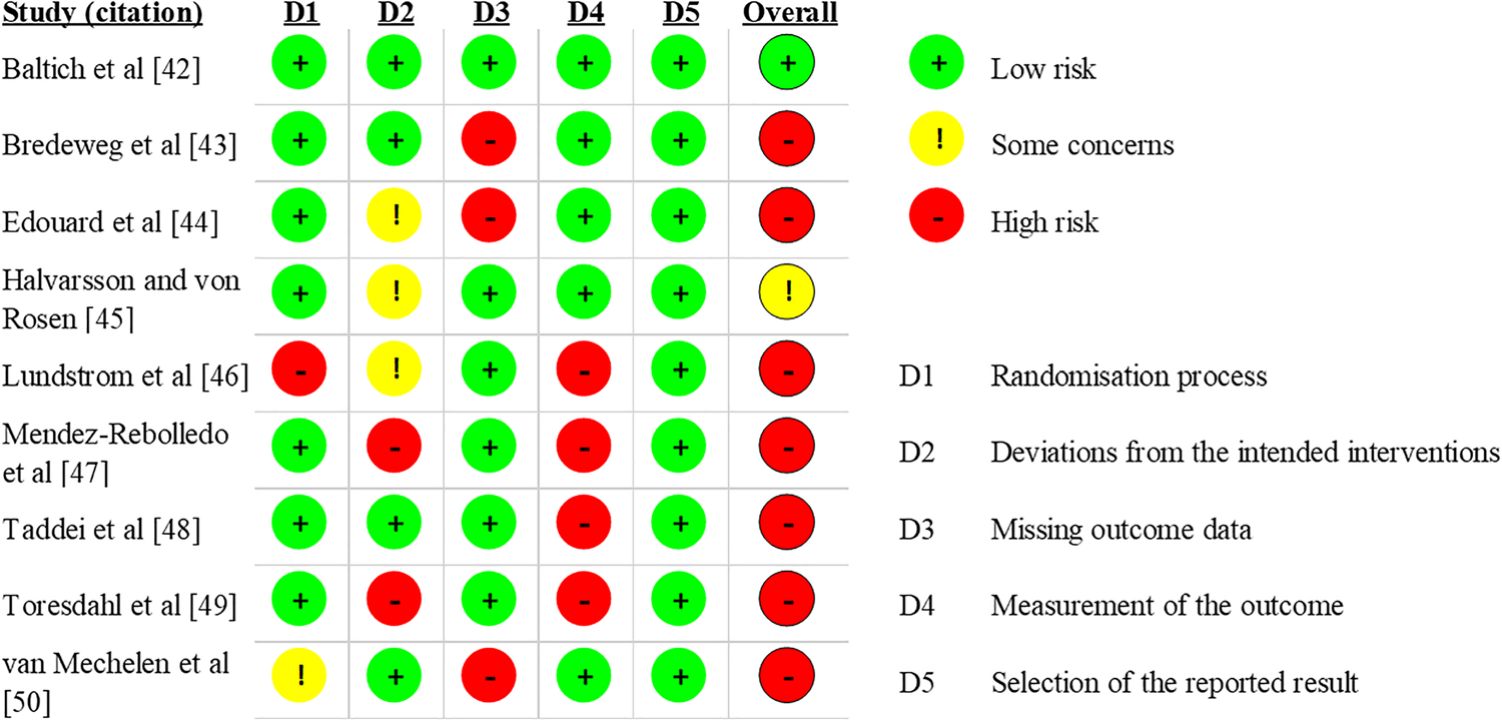
Outcomes of the risk of bias assessment

Figure 2 shows the outcomes of the risk of bias assessment. Among the nine included studies, seven had high overall risk of bias [43, 44, 46–50], one was with some concerns [45], and one had low overall risk of bias [42]. Common reasons for high risk of bias included participants and/or carers being aware of the assignment of the intervention group during the delivery of intervention (D2); outcome data not available for some randomized participants due to dropout (D3); and outcome assessors (usually participants themselves in self-reporting injury) being aware of the intervention received by participants (D4). GRADE evidence profile showed that the ‘injury risk’ outcome containing seven of the nine studies had moderate certainty of evidence while the ‘injury rate’ outcome containing six of the nine studies had high certainty of evidence (ESM Tables S3 and S4).

### Meta-Analysis

**Fig. 3:**
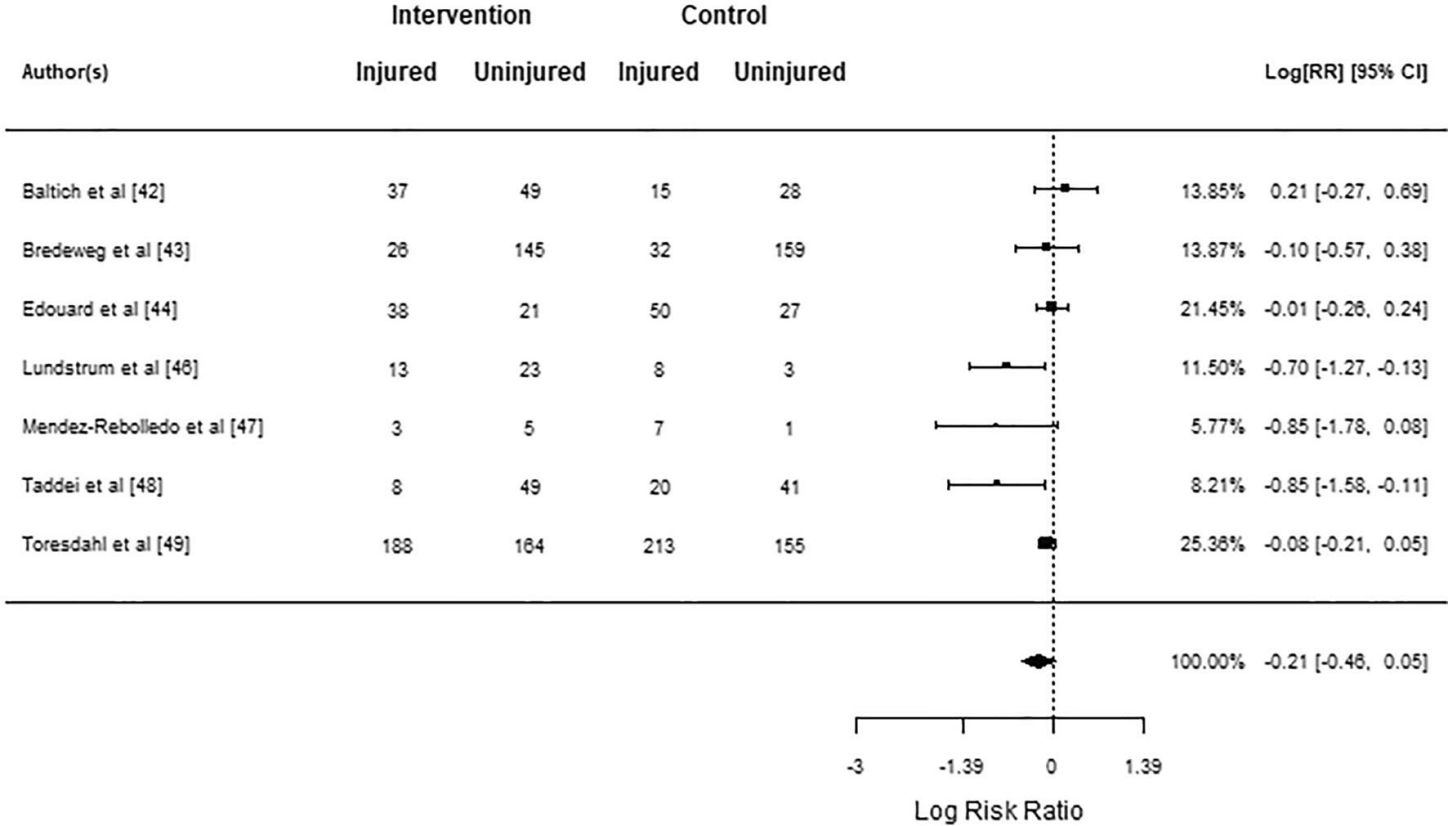
Injury risk meta-analysis forest plot showing the observed outcomes and the estimate of the random-effects model

**Fig. 4:**
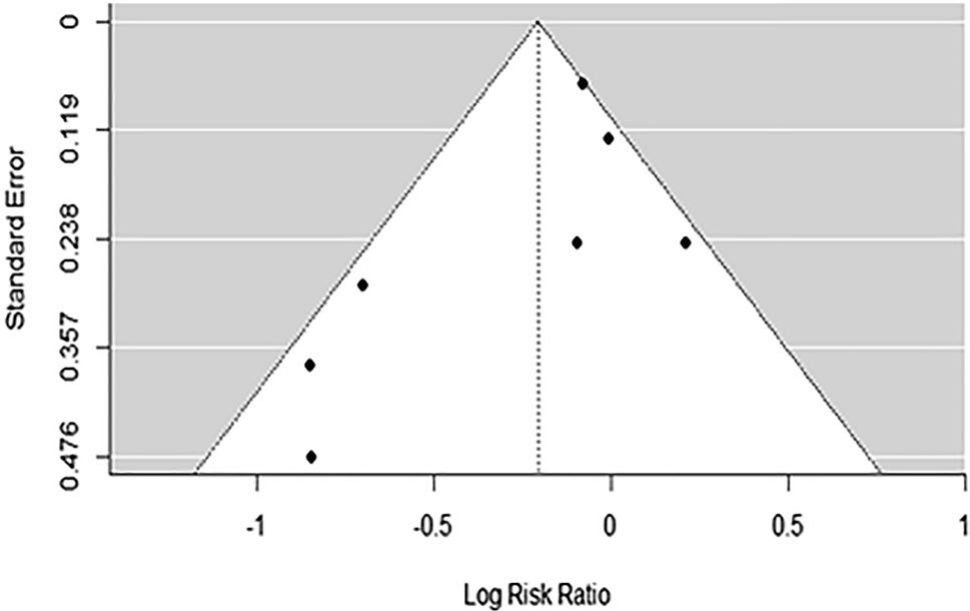
Injury risk funnel plot

Table 3 summarizes the outcomes of each study. The meta-analysis for injury risk included seven studies [42–44, 46–49], of which two had negative log risk ratios (95% CI not overlapping zero), meaning that the intervention group had a lower injury risk compared to the control group. Only Baltich et al. [42] showed a positive log risk ratio of 0.21; however, the 95% CI overlapped zero. The average log risk ratio was − 0.21 (95% CI; − 0.46 to 0.047), which was a statistically non-significant effect (*z* = − 1.60; *p* = 0.110; Fig. 3) with moderate quality of evidence (downgraded 1 level due to risk of bias). *Q*-test showed significant heterogeneity between true outcomes ($Q\left(6\right)=13.00$, $p=0.043$, ${\widehat{\tau }}^{2}=0.061$, ${I}^{2}=65.02$%; 95% CI − 0.75 to 0.34). Standardized residuals revealed no outliers and Cook’s distances revealed no overly influential studies. A funnel plot is shown in Fig. 4, with linear regression indicating plot asymmetry (*p* = 0.044); however the rank correlation test showed no significant asymmetry (*p* = 0.381).

**Table 3:** Outcomes of studies

Study	Compliance	Definition of injury	Injuries clinically diagnosed?	Injury incidence/prevalence	Number of recorded injuries	Other injury metrics reported	Other injury metric results
Baltich et al. [42]	RT: 93% ≥ 2 sessions per week FSST: 86% ≥ 2 sessions per week Stretch: 86% ≥ 2 sessions per week	Restriction in running > 1 week	Yes	RT: 27.7/1000 h (95% CI 15.1–46.5) FSST: 25.3/1000 h (95% CI 14.7–40.5) Stretch: 22.4/1000 h (95% CI 11.6–39.2)	56	Injury severity	NSD
Bredeweg et al. [43]	≥ 10 sessions: 71.6% 7–9 sessions: 14.2% 4–6 sessions: 6.6% No sessions: 7.6%	Restriction in running > 1 week	No	Precon: 31/1000 h (95% CI 24.0–38.0) Con: 32/1000 h (24.0–37.0)	58	–	–
Edouard et al. [44]	6 of 59 participants compliant	Pain, physical complaint or musculoskeletal lesion sustained during participation in training or competition, regardless of whether it received medical attention or its consequences with respect to impairments in connection with competition or training	No	Int: 38 participants ≥ 1 injury Con: 50 participants ≥ 1 injury Six compliant with intervention, 3 injured and 3 uninjured	≥ 88 (88 participants experienced injuries, while multiple injuries on the same person were not counted)	–	–
Halvarsson & von Rosen [45]	Mean 2.2 ± 0.6 times/week; 63% ≥ 2 times per week, 37% < 2 times per week	Any physical complaint resulting in moderate or severe reductions in training volume, or moderate or severe reduction in performance, or complete inability to participate	No (Oslo Sports Trauma Research Center Overuse Injury Questionnaire)	Int: 9.4/1000 h (SD: 3.6; 95% CI 8.1–10.7) Con: 11.2/1000 h (SD: 3.9; 95% CI 9.8–12.6)	64	Mean difference (control vs. intervention) in injury prevalence (%) by engagement with exercises	CE < 2/week: − 0.1% (OR: 0.88, 95% CI 0.35–2.25, *p* = 0.792) CE ≥ 2/week: − 7.9% (OR: 0.26, 95% CI 0.07–0.92, ***p* = 0.037**)
Lundstrom et al. [46]	Average days missed not due to injury (total training days 48–60): Core: 1.5 Plyo: 0.5 Con: 0.4	Training days missed due to injury	No (self-report training log)	Days missed: Core: 2.7 ± 5.1 Plyo: 1.2 ± 4.9 Con: 4.1 ± 2.9 *p* value (group) = 0.103	16		
Mendez-Rebolledo et al. [47]	100% adherence	Participant unable to fully take part in the training session	Yes	Con: 17.7/1000 h (SD: 1.4; 95% CI 9.4–25.9) Int: 4.2/1000 h (SD: 0.8; 95% CI: 0.2–8.2)	29		
Taddei et al. [48]	Int: 88% adherence (90.4% in first 8 weeks, 83.5% weeks 8–16, 68.5% weeks 16–24, 62.5% weeks 24–32, 48.9% weeks 32–40) Stretch: not reported	Musculoskeletal pain or injury caused by running practice that induces changes in the form, duration, intensity, or frequency of training for at least 1 week	Yes	Con: 20 participants (16.9%; 95% CI 10.2–23.7) Int: 8 participants (6.7%, 95% CI 2.2–11.3%)	28 (participants were dropped out after experiencing first injury)	Kaplan–Meier survival estimate between-group log-rank test Cox proportional hazards analysis	Log rank test: ***p* = 0.027** Hazard ratio con were 2.42 × more likely to experience injury after 1 year (***p* = 0.035,** 95% CI 1.98–3.62) Each year of age associated with 1.07 × higher likelihood of injury (p = 0.015)
Toresdahl et al. [49]	2.0 ± 1.2 sessions per week performed 176 (50%) participants performed average ≥ 2 times per week	Injury limiting training	No (self-report survey)	Major injuries: Con: 27 Int: 25 (RR: 0.97; 95% CI 0.57–1.63, *p* = 0.90) Minor injuries: Con: 186 Int: 163 (RR: 0.92; 95% CI 0.79–1.07, *p* = 0.26)	349	Compliant (≥ 2 times per week completion) vs. non-compliant	Compliant participants sub-analysis for minor injuries: compliant 41.5% vs. non-compliant 56.2%, *p* = 0.01. Major injuries: compliant 9.1% vs. non-complaint 8.0%, NSD
Van Mechelen et al. [50]	Warm-up as prescribed 68% Cool-down as prescribed 65% Stretching as prescribed 47% No compliance warm-up 8%, cool-down 11%, stretching 40% (large % of control group performed warm-up, cool-down and stretching but largely not comparable to prescription)	(1) Participant stopped running, (2) participant could not run on the next occasion, (3) participant could not go to work the next day, (4) participant needed medical attention, or (5) participant suffered from pain or stiffness during 10 subsequent days while running	No (self-report diary)	Con: 4.9/1000 h (95% CI 3.1–7.4) Int: 5.5/1000 h (95% CI 3.6–8.0) NSD	49	Analysis by sub-categories based upon interactions of weekly running distance, age, knowledge on prevention of injury Between-group differences and location of injury	NSD NSD

*RT* resistance training, *FSST* functional sport-specific strength training, *CI* confidence interval, *NSD* not statistically different, *Precon* pre-conditioning intervention, *Con* control group, *SD* standard deviation, *Int* Intervention group, *CE* completed exercise frequency, *OR* odds ratio, *plyo* plyometric, *RR* risk ratio

**Fig. 5:**
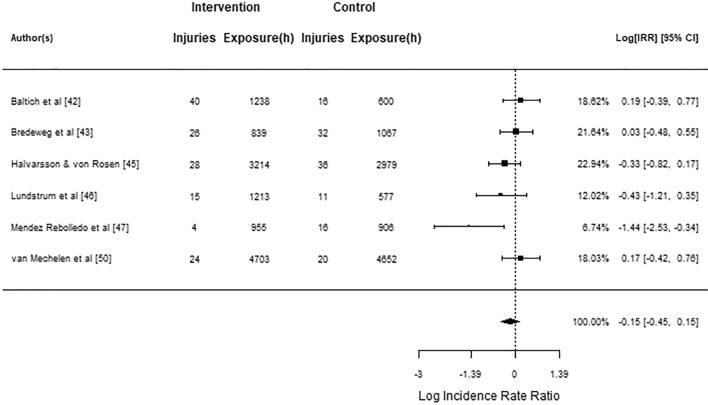
Injury rate meta-analysis forest plot showing the observed outcomes and the estimate of the random-effects model

The injury rate meta-analysis included six studies [42, 43, 45–47, 50]. One investigation showed a negative log incidence rate ratio [47] and others did not differ from zero. The pooled log incidence rate ratio was − 0.15 (95% CI − 0.45 to 0.15), which was not statistically significant (*z* = − 0.98; *p* = 0.329; Fig. 5) with high quality of evidence. *Q*-test revealed no significant heterogeneity between true outcomes ($Q\left(5\right)=9.23$, $p=0.100$, ${\widehat{\tau }}^{2}=0.04$, ${I}^{2}=28.50$%; 95% CI − 0.65 to 0.34). No outliers were identified, and Cook’s distance revealed no studies skewing the result.

**Fig. 6:**
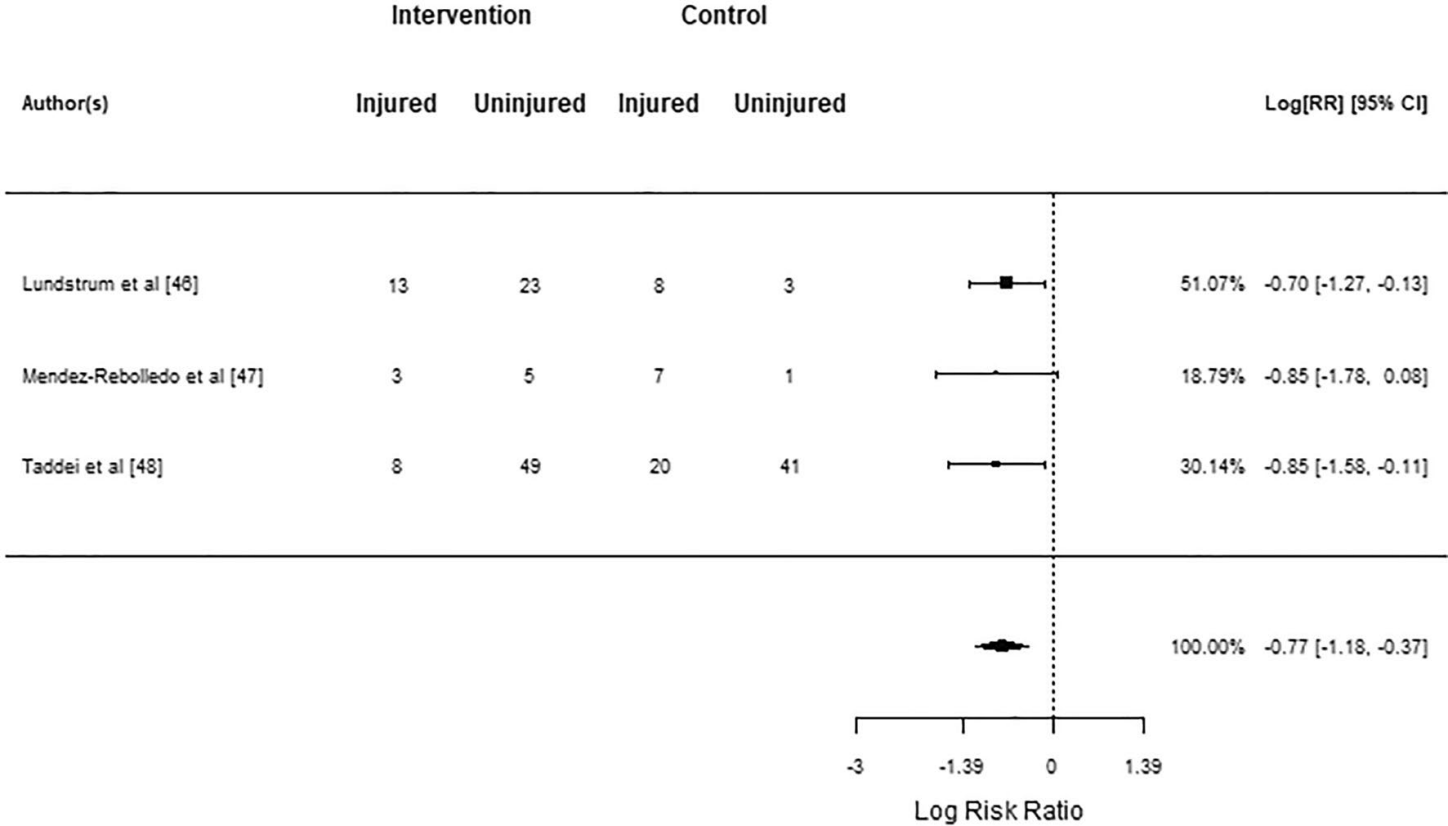
Post hoc injury risk meta-analysis forest plot showing the observed outcomes and the estimate of the random-effects model

The post hoc injury risk meta-analysis was performed on the three studies that used supervision [46–48]. Two studies showed negative log risk ratios that did not differ from zero [46, 48], and the pooled log risk ratio of − 0.77 (95% CI − 1.18 to − 0.37) was statistically significant in favor of the intervention (*z* = − 3.75, *p* < 0.001; Fig. 6). No significant heterogeneity between true outcomes were found by Q test ($Q\left(2\right)=0.13$, $p=0.938$, ${\widehat{\tau }}^{2}=0.00$, ${I}^{2}=0.00$%). Studentized residuals and Cook’s distances revealed no outliers or overly influential studies.

## Discussion

The aim of this study was to review and quantify the effect of non-running-based exercise programs on the risk of RRIs in endurance runners. When data were pooled together, exercise-based injury prevention programs provided no significant positive effect on either injury risk or injury rate. However, an interesting finding was that a significant positive effect on injury risk in favor of the exercise intervention group was noted when only exercise interventions with an element of supervision were included in the analysis. These findings suggest that exercise interventions designed to reduce the risk of RRI are unlikely to be successful unless completed under supervision, which is likely due to increased compliance with the program.

Similar reviews on this topic are now outdated [19], cover multi-sport participation [27, 29, 30], or did not adequately define the proportion of training that constituted running in the participant population [19, 20]. This review provides an update, including six studies published in the last 4 years, using more stringent inclusion criteria, and includes meta-analyses on both injury rate and injury incidence. The findings of this paper largely confirm those of previous reviews [19, 20] in less well-defined endurance running populations (athletes from running-based sports), that there is little evidence to support inclusion of S&C exercises for the purpose of reducing RRIs. However, compared to the positive risk ratio found by Yeung et al. [19] in their conditioning exercises intervention result (1.20), and the risk ratio of 0.98 found by Kozinc et al. [20] for movement therapy-based interventions, the log risk ratio (− 0.21) and log incidence ratio (− 0.15) we observed are numerically more in favor of an exercise intervention. In contrast with these findings, the review by Lauersen and co-workers [29] concluded that multi-modal exercise interventions, particularly strength training, offered a significant protective effect against overuse injury in athletes. The six studies that contributed to the meta-analysis by Lauersen et al. [29] were largely from game-sports, several of which included throwing skills, and half used adolescent athletes. Although overlap exists between the types of overuse injury reported in games sport athletes and endurance runners, the mechanisms that underpin these injuries may differ [51]. For example, loading on lower limb musculoskeletal structures tends to be higher and more varied in games sports, but is lower in magnitude and distribution in endurance running. Thus, the protective effect provided by an adjunct exercise intervention may also vary.

Overall risk of bias was high in the studies included (seven of nine studies classified ‘high risk’), indicating that the quality of research in this area is currently poor. Due to the nature of the intervention and the characteristics of the target population, eliminating bias in some domains is problematic. For instance, in a running community or club setting, it is often difficult to blind participants to each other’s intervention. Furthermore, a study with low compliance with an intervention will likely have a high percentage of dropouts who subsequently do not contribute injury data, generating a high risk of bias in domain #3. Six studies asked participants to self-report injuries [43–46, 49, 50], meaning the outcome assessor was the participant themself. Participants’ interpretation of what constitutes an ‘injury’ may therefore have influenced results in these studies. Furthermore, participants’ expectations and beliefs concerning the role of non-running-based exercise interventions in the prevention of injury may have influenced reporting and study outcomes [52]. Only one study was considered to have a low risk of bias [42], and it could be used as an example for future studies in this area to replicate. Despite the high risk of bias, certainty of evidence for both injury risk and injury rate were moderate and high respectively, mainly because all studies were randomized controlled trials and no serious issues in consistency, directness, and precision were identified.

The three studies that provided supervision reported the lowest log risk ratios and log incidence risk ratios in both the injury risk and injury rate meta-analyses [46–48]. However, it is worth noting that these studies had small sample sizes (16 ≤ *n* ≤ 118) and thus relatively higher standard errors compared to other studies. Consequently, a post hoc meta-analysis was performed on these studies. The results showed a statistically significant difference favoring the intervention with a lower log risk ratio compared to the original injury risk meta-analysis (− 0.77, *p* < 0.001). Interestingly, a previous study in military cadets also reported that a group performing a ‘dynamic integrated movement enhancement’ warm-up under professional supervision had a significantly lower injury incidence compared to a group who complied with the same program but with minimal supervision [53]. Further, another study in military recruits reported lower incidence of overuse anterior knee pain following a closely supervised strength training and stretching warm-up intervention compared to a group following a traditional warm-up approach [54].

Two studies observed a significant protective effect of the intervention on injury occurrence [47, 48]. These studies reported high adherence to the intervention program compared to other studies, which may indicate that it is compliance with the injury prevention exercise regimen that is important, rather than supervision per se. Indeed, sub-analysis in one other study in this review reported that the exercise intervention provided a protective effect against overuse injury only in participants who complied with the program [45]. It is also possible that participants who adhered to the exercise program also exhibited other healthy behaviors associated with lower injury risk, such as better nutrition [55] and lifestyle habits [56]; however these factors were not reported in any study. Poor adherence to the exercise intervention may therefore partly explain the lack of significant effect across the other studies reviewed. In studies including games players that reported a positive impact of an exercise-based injury prevention program on overuse injuries, adherence was high (≥ 77%) [57–59]. A dose–response relationship has also been noted for exposure to neuromuscular training and reduction of sport-related injury risk in youth athletes [28], meaning future studies should prioritize supervision as part of study design and administration, which is likely to maximize adherence to the intervention and thus physiological adaptation. Based upon these findings, a supervised injury prevention exercise session performed two to four times per week is most likely to reduce risk of a RRI [45, 47, 48].

Most studies included participants who were considered novice or recreational level runners [42, 43, 46, 49, 50]. The level of compliance with the intervention in these studies was typically poor, whereas the two studies noting a protective effect against injury reported on high performing adolescent runners [47] or runners with an average experience of ~ 6 years [48]. In general, it appears that more well-trained or experienced runners engage with injury prevention protocols to a greater extent compared to their lesser trained counterparts. This observation is also in-line with strength training participation trends in the running community, with a higher percentage of international and national standard runners regularly using resistance training and plyometrics compared to local club standard runners [16].

A wide range of exercise modalities were used as part of the interventions that found a positive effect on injury incidence [45, 47, 49]. However, the programs in these investigations did not differ markedly from the types of exercises prescribed in studies observing no difference in any injury outcomes [42–44, 46, 49, 50]. The exception was the study by Taddei et al. [48] that used strengthening exercises specifically for the foot–ankle muscles, which the authors speculated would improve the structure and function of the foot, thus attenuating the loads runners experience during the stance phase of gait. The study observed a significantly lower rate of RRIs compared to a running plus static stretching control group by a factor of 2.42 [48]. Based on the success of this intervention in reducing the rate of RRIs, future studies should include similar programs of foot–ankle strengthening to further explore the efficacy of this novel conditioning approach. Given that adherence to a program appeared to determine its effectiveness, it is currently not possible to provide more specific recommendations on the most appropriate types of exercise modalities to prevent injury in runners.

There is strong evidence surrounding the value of supplementary training to improve neuromuscular performance as a strategy to reduce injury risk in team and youth sports [27–30, 60–62]. Despite low-quality evidence, neuromuscular and resistance training are also recommended to reduce injury risk in military populations [63]. The aforementioned reviews were based upon far higher participant numbers (*n* = 13,355–32,254 from 10 to 25 studies) compared to this review (*n* = 1904 from nine studies) indicating that more research is required specifically in endurance runners using larger sample sizes. Given the differences that exist in injury types and mechanisms between game sports and endurance running, it is currently not reasonable to use the recommendations from other athlete populations and apply these to runners. Resistance training or multi-component neuromuscular training (including strength, core stability, balance, plyometric and speed/agility exercises) were not used in several studies that failed to demonstrate a protective effect of an injury prevention program [43, 46, 50], suggesting that the content of these interventions may not have been appropriate. In particular, higher volumes and intensities of strength training have demonstrated consistently favorable results for overuse injury outcomes in other sports [30], yet only four studies in this review utilized strength-based exercises, and these were of relatively low volume and intensity [42, 44, 47, 49]. Thus, further research is warranted on the effect of strength training on RRI risk in endurance runners. Studies that target increasing the resilience of clinically relevant structures that are vulnerable to injury in runners are also currently lacking. This was the approach taken by Taddei and colleagues [48], which utilized an exercise intervention for the feet; however the largest differences in injury incidence between the intervention and control group were at the knee and thigh. Given the repetitive and localized nature of loading on musculoskeletal structures during endurance running, exploring exercise interventions that strengthen specific tissues should form an important avenue of future research.

An important feature of injury related studies is the assessment and diagnosis of injuries by a medical professional. In four of the nine studies, injuries were self-reported by participants and were not clinically diagnosed [43–46]. This creates an important source of bias because participants may be less consistent in evaluating incidence and extent of injuries than trained clinicians. Further, the definition of an overuse injury differed between studies, which is also likely to have influenced results. Participants in three studies were classified as being injured when they restricted their running training for over a week [42, 43, 48], whereas other studies defined injury as a single missed training run [46, 47, 50] or impairment of one or more running sessions [44, 45, 49].

This review has several important strengths that give credibility to the results. Firstly, the inclusion criteria employed in this review were more stringent than in previous reviews, increasing the internal validity of the findings. Bias was minimized in the systematic review process by having two authors independently screen studies and extract data. Finally, GRADE evidence profiles were generated to evaluate the level of certainty in the results obtained for the two main outcome measures, highlighting that the quality of evidence is moderate for the injury risk findings, and high for the injury rate outcomes. This paper also has limitations, which should be recognized. Intention-to-treat data were used in meta-analyses where possible but several studies that did not report intention-to-treat data were also included, which creates a potential source of bias. When performing the meta-analysis on injury risk, length of an intervention and follow-up duration were also not accounted for. Since the duration of interventions and follow-up varied widely across studies (from 6 weeks to 12 months), attributing equal weight to all studies may underestimate the weighting of longer interventions.

## Directions for Future Research

Based on the aforementioned discussion, it is recommended that future research in this area:

- Uses supervised exercise interventions. Based on the post hoc meta-analysis results, it is possible that supervision is necessary for exercise-based intervention programs to achieve beneficial effect with reducing risks of RRIs, and more research evidence is therefore needed with supervised interventions for conclusions to be drawn.
- Sets the frequency of the intervention at two to four times per week. As most of the existing studies apply interventions two to four times per week, it is recommended that future studies follow this session frequency so comparisons can be made.
- Examines whether traditional strength training reduces injury occurrence. This is proposed because the existing studies vary a great deal in their applied exercise modalities while traditional strength training, which has shown effectiveness in reducing injury occurence in other settings, has seldom been used in studies with endurance runners. Traditional strength training is also one of the most widely used exercise-based interventions in practice so more research is needed to separately investigate this exercise modality as a potential strategy to reduce injury risk.
- Further evaluates the efficacy of ‘foot-core training’ (i.e., foot and ankle-based conditioning) and other targeted exercises as a novel injury prevention approach. This novel approach was used in one study and a significant effect was found on reducing the incidence of RRIs. More research evidence is needed to determine whether this promising effect can be reproduced in different cohorts.

## Conclusions

In conclusion, there is currently insufficient evidence to recommend the use of exercise-based prevention strategies to reduce the risk of RRIs in endurance runners. Studies to date have largely been low-quality and used small sample sizes, and more well-designed randomized controlled trials are therefore needed in the future. This review underlines the importance of supervision when evaluating the effect of exercise interventions on reducing RRIs. Studies that had minimal or no supervision also tend to have poor compliance, which diminishes the exposure to the intervention and reduces the likelihood of a positive result. Studies that observed favorable results for overuse injury outcomes used multicomponent neuromuscular training (strength, agility, balance, core, plyometrics, bodyweight exercises), or targeted exercises for the foot and ankle, on two to four occasions per week for longer than 6 weeks.

### Supplementary Information

Below is the link to the electronic supplementary material.

Supplementary file1 (DOCX 97 KB)

## Declarations

### Data availability statement

The authors declare that the data supporting the findings of this study can be found within this paper and in Electronic Supplementary Material Table S5. Should any data be needed in an alternative format, they can be provided by the corresponding author upon reasonable request.
