# Supplementary material for: Do Exercise-Based Prevention Programs Reduce Injury in Endurance Runners? A Systematic Review and Meta-Analysis
Source: Sports Med. 2024 Jan 23;54(5):1249–67. doi: 10.1007/s40279-024-01993-7 (PMC11127851; doi:10.1007/s40279-024-01993-7)
Supplement: Supplementary file 1 — Supplementary file1 (DOCX 97 KB) [file 40279_2024_1993_MOESM1_ESM.docx]

**Electronic Supplementary Materials**

**Electronic Supplementary Appendix S1. Systematic Search Key Words**

**PubMed Search**

((("prevent"[Title/Abstract] OR "preventative"[Title/Abstract] OR "preventatively"[Title/Abstract] OR "preventatives"[Title/Abstract] OR "prevented"[Title/Abstract] OR "preventing"[Title/Abstract] OR "prevention and control"[Title/Abstract] OR ("prevention"[Title/Abstract] AND "control"[Title/Abstract]) OR "prevention and control"[Title/Abstract] OR "prevention"[Title/Abstract] OR "prevention s"[Title/Abstract] OR "preventions"[Title/Abstract] OR "preventive"[Title/Abstract] OR "preventives"[Title/Abstract] OR "prevents"[Title/Abstract] OR (("lower"[Title/Abstract] OR "lowered"[Title/Abstract] OR "lowering"[Title/Abstract] OR "lowers"[Title/Abstract]) AND ("risk"[Title/Abstract] OR "risk"[Title/Abstract])) OR (("lower"[Title/Abstract] OR "lowered"[Title/Abstract] OR "lowering"[Title/Abstract] OR "lowers"[Title/Abstract]) AND ("likelihoods"[Title/Abstract] OR "probability"[Title/Abstract] OR "probability"[Title/Abstract] OR "likelihood"[Title/Abstract])) OR (("lower"[Title/Abstract] OR "lowered"[Title/Abstract] OR "lowering"[Title/Abstract] OR "lowers"[Title/Abstract]) AND ("occur"[Title/Abstract] OR "occurance"[Title/Abstract] OR "occurence"[Title/Abstract] OR "occurences"[Title/Abstract] OR "occuring"[Title/Abstract] OR "occurring"[Title/Abstract] OR "occurs"[Title/Abstract])) OR ("reduce"[Title/Abstract] OR "reduced"[Title/Abstract] OR "reduces"[Title/Abstract] OR "reducing"[Title/Abstract]) OR ("improve"[Title/Abstract] OR "improved"[Title/Abstract] OR "improvement"[Title/Abstract] OR "improvements"[Title/Abstract] OR "improves"[Title/Abstract] OR "improving"[Title/Abstract] OR "improvment"[Title/Abstract]) OR ("decrease"[Title/Abstract] OR "decreased"[Title/Abstract] OR "decreases"[Title/Abstract] OR "decreasing"[Title/Abstract]))

AND

("injurie"[Title/Abstract] OR "injuries"[Title/Abstract] OR "injuries"[Title/Abstract] OR "injury s"[Title/Abstract] OR "injuryed"[Title/Abstract] OR "injurys"[Title/Abstract] OR "injury"[Title/Abstract] OR "cumulative trauma disorders"[MeSH Terms] OR "fractures stress"[Title/Abstract] OR "fractures bone"[Title/Abstract] OR "patellofemoral pain syndrome"[Title/Abstract] OR ("anterior"[Title/Abstract] AND ("knee"[Title/Abstract] OR "knee joint"[Title/Abstract] OR ("knee"[Title/Abstract] AND "joint"[Title/Abstract]) OR "knee joint"[Title/Abstract]) AND "pain"[Title/Abstract]) OR "iliotibial band syndrome"[Title/Abstract] OR "medial tibial stress syndrome"[Title/Abstract] OR "tendinopathy"[Title/Abstract] OR "tendinopathy"[Title/Abstract] OR "tendinopathy"[Title/Abstract] OR "fasciitis plantar"[Title/Abstract])

AND

("program"[Title/Abstract] OR "program s"[Title/Abstract] OR "programe"[Title/Abstract] OR "programes"[Title/Abstract] OR "programme"[Title/Abstract] OR "programme s"[Title/Abstract] OR "programmes"[Title/Abstract] OR "programs"[Title/Abstract] OR ("intervention s"[Title/Abstract] OR "interventions"[Title/Abstract] OR "interventive"[Title/Abstract] OR "intervention"[Title/Abstract]) OR "home-based"[Title/Abstract] OR "supplementary"[Title/Abstract] OR ("conditioning"[Title/Abstract] OR "conditionings"[Title/Abstract]) OR ("preconditioned"[Title/Abstract] OR "preconditioning"[Title/Abstract] OR "preconditioning s"[Title/Abstract] OR "preconditionings"[Title/Abstract]) OR "warmup"[Title/Abstract] OR ("warm up exercise"[Title/Abstract] OR ("warm up"[Title/Abstract] AND "exercise"[Title/Abstract]) OR "warm up exercise"[Title/Abstract] OR ("warm"[Title/Abstract] AND "up"[Title/Abstract]) OR "warm up"[Title/Abstract]) OR "pre-exercise"[Title/Abstract] OR "preexercise"[Title/Abstract] OR ("strength"[Title/Abstract] OR "strengths"[Title/Abstract]) OR ("resistance"[Title/Abstract] OR "resistants"[Title/Abstract] OR "resisted"[Title/Abstract] OR "resistence"[Title/Abstract] OR "resistent"[Title/Abstract]) OR ("loaded"[Title/Abstract] OR "loading"[Title/Abstract] OR "loadings"[Title/Abstract] OR "loads"[Title/Abstract]) OR "jump"[Title/Abstract] OR ("jumping"[Title/Abstract] OR "jumpings"[Title/Abstract] OR "jumps"[Title/Abstract]) OR ("plyometric"[Title/Abstract] OR "plyometrics"[Title/Abstract]) OR ("exercise"[Title/Abstract] OR "exercises"[Title/Abstract] OR "exercise therapy"[Title/Abstract] OR ("exercise"[Title/Abstract] AND "therapy"[Title/Abstract]) OR "exercise therapy"[Title/Abstract] OR "exercise s"[Title/Abstract] OR "exercising"[Title/Abstract]) OR ("flexibilities"[Title/Abstract] OR "flexibility"[Title/Abstract]) OR ("stretch"[Title/Abstract] OR "stretches"[Title/Abstract] OR "stretching"[Title/Abstract] OR "stretchings"[Title/Abstract]) OR ("balance"[Title/Abstract] OR "balanced"[Title/Abstract] OR "balances"[Title/Abstract] OR "balancing"[Title/Abstract]) OR ("unstability"[Title/Abstract] OR "unstable"[Title/Abstract]))) OR ("pilate"[Title/Abstract] OR "pilates"[Title/Abstract]) OR ("core stability"[Title/Abstract] OR ("core"[Title/Abstract] AND "stability"[Title/Abstract]) OR "core stability"[Title/Abstract]))

AND

("running"[Title/Abstract] OR "running"[Title/Abstract] OR "runnings"[Title/Abstract] OR ("runner"[Title/Abstract] OR "runner s"[Title/Abstract] OR "runners"[Title/Abstract]) OR "endurance"[Title/Abstract] OR "marathon running"[Title/Abstract] OR "track and field"[Title/Abstract] OR ("militaries"[Title/Abstract] OR "military personnel"[Title/Abstract] OR ("military"[Title/Abstract] AND "personnel"[Title/Abstract]) OR "military personnel"[Title/Abstract] OR "military"[Title/Abstract] OR "military s"[Title/Abstract]) OR ("military personnel"[Title/Abstract] OR ("military"[Title/Abstract] AND "personnel"[Title/Abstract]) OR "military personnel"[Title/Abstract] OR "soldier"[Title/Abstract] OR "soldiers"[Title/Abstract] OR "soldier s"[Title/Abstract]) OR "military personnel"[Title/Abstract] OR "army"[Title/Abstract])

AND

("controling"[All Fields] OR "controlling"[All Fields] OR "controls"[All Fields] OR "control"[All Fields] OR "control groups"[MeSH Terms] OR ("control"[All Fields] AND "groups"[All Fields]) OR "control groups"[All Fields] OR "controlled"[All Fields] OR ("comparison"[All Fields] OR "comparisons"[All Fields]))

**Web of Science Search**

TS=((“prevent*” OR “reduc*” OR “lower risk” OR "lower likelihood" OR "lower occurrence" OR “improve*” OR “decreas*”) AND (“injur*” OR “cumulative trauma disorder” OR “repetitive strain” OR “repetitive stress” OR “stress injury” OR “stress fracture” OR “bone stress” OR “fractures, bone” OR “patellofemoral pain syndrome” OR “anterior knee pain” OR “Iliotibial Band Syndrome” OR “medial tibial stress syndrome” OR “tendin*” OR “fasciitis, plantar”) AND (“program*” OR “intervention” OR “home-based” OR “supplementary” OR conditioning OR preconditioning OR “warm-up” OR “warmup” OR “warm up” OR “pre-exercise” OR “preexercise” OR strength OR resistance OR “core” OR jump OR “jumping” OR plyometric* OR “circuit-based” OR exercise OR flexibility OR stretching) AND (“runn*” OR endurance OR “middle-distance” OR “long-distance” OR marathon OR “ultra-distance” OR “ultra-marathon” OR “cross-country” OR “cross country” OR “track and field” OR military OR soldiers OR “armed forces” OR concurrent))

AND

ALL=(control OR controlled OR comparison)

**SportDiscus Search**

English; SU subjects; academic journals and dissertations

"wounds & injuries” OR “overuse injuries” “stress fractures” OR “bone fractures” OR “PLICA syndrome” OR “anterior compartment syndrome” OR “Iliotibial Band Syndrome” OR “shin splints” OR "tendinopathy" OR “tedinosis” OR "tendinitis" OR “plantar fasciitis” OR “running injuries”

AND

"physical training & conditioning" OR "therapeutics" OR "occupational therapy" OR "physical activity" OR "physical fitness" OR "warmup" OR "strength training" OR "resistance training" OR "jumping training" OR "plyometrics" OR “circuit training” OR "exercise" OR “stretch” or “pilates method” OR “concurrent training”

AND

"runners" OR "running" OR "endurance sports" OR “endurance athletes” OR “endurance sports training” OR “middle-distance running” OR “long-distance runners” OR “long-distance running” OR “long-distance relay races” OR "marathons" OR “marathon running” OR “marathon running training” OR “ultramarathon running” OR “ultraendurance sports” OR “ultramarathon running training” OR “cross-country runners” OR “cross-country running” OR "trail running" OR “track and field” OR "military" OR "soldiers" OR “armed forces” OR "orienteer" OR "orienteering"

**Electronic Supplementary Appendix S2. Codes for Meta-analysis in R**

# Import the metafor package and readxl

> library(metafor)

> library(readxl)

# Import raw data from computer (see section 2)

> injury_rate_data <- read_excel("C:/Users/Inspiron/OneDrive - Loughborough University/Desktop/PhD/articles/systematic review/meta-analysis/injury rate data.xlsx")

> injury_incidence_data <- read_excel("C:/Users/Inspiron/OneDrive - Loughborough University/Desktop/PhD/articles/systematic review/meta-analysis/injury incidence data.xlsx")

> post_hoc_injury_rate_data <- read_excel("C:/Users/Inspiron/OneDrive - Loughborough University/Desktop/PhD/articles/systematic review/meta-analysis/post-hoc injury rate data.xlsx")

# Note: Injury_rate corresponds with injury risk in the study. Injury_incidence corresponds with injury rate in the study. The names were changed after the codes were written.

# Calculations

> injury_rate <- escalc(measure="RR", ai=tpos, bi=tneg, ci=cpos, di=cneg, slab=paste(author, year, sep=" "), data=injury_rate_data)

> res_injury_rate <- rma(yi, vi, data=injury_rate)

> injury_incidence <- escalc(measure="IRR", x1i=tcount, t1i=texpo, x2i=ccount, t2i=cexpo, slab=paste(author, year, sep=" "), data=injury_incidence_data)

> res_injury_incidence <- rma(yi, vi, data=injury_incidence)

> post_hoc_injury_rate <- escalc(measure="RR", ai=tpos, bi=tneg, ci=cpos, di=cneg, slab=paste(author, year, sep=" "), data=post_hoc_injury_rate_data)

> res_post_hoc_injury_rate <- rma(yi, vi, data=post_hoc_injury_rate)

# Injury risk forest plot

> forest(res_injury_rate, showweights=TRUE, at=log(c(.05, .25, 1, 4)), xlim=c(-16,6),

+ ilab=cbind(tpos, tneg, cpos, cneg), ilab.xpos=c(-10,-8,-6,-4),

+ cex=.75, header="Author(s)", mlab="")

> op <- par(cex=.75, font=2)

> text(c(-10,-8,-6,-4), res_injury_rate$k+2, c("Injured", "Uninjured", "Injured", "Uninjured"))

> text(c(-9,-5), res_injury_rate$k+3, c("Intervention", "Control"))

> par(op)

# Injury rate forest plot

> forest(res_injury_incidence, showweights=TRUE, at=log(c(.05, .25, 1, 4)), xlim=c(-16,6),

+ ilab=cbind(tcount, texpo, ccount, cexpo), ilab.xpos=c(-10,-8,-6,-4),

+ cex=.75, header="Author(s)", mlab="")

> op <- par(cex=.75, font=2)

> text(c(-10,-8,-6,-4), res_injury_incidence$k+2, c("Injuries", "Exposure(h)", "Injuries", "Exposure(h)"))

> text(c(-9,-5), res_injury_incidence$k+3, c("Intervention", "Control"))

> par(op)

# Post-hoc injury risk meta-analysis forest plot

> forest(res_post_hoc_injury_rate, showweights=TRUE, at=log(c(.05, .25, 1, 4)), xlim=c(-16,6),

+ ilab=cbind(tpos, tneg, cpos, cneg), ilab.xpos=c(-10,-8,-6,-4),

+ cex=.75, header="Author(s)", mlab="")

> op <- par(cex=.75, font=2)

> text(c(-10,-8,-6,-4), res_post_hoc_injury_rate$k+2, c("Injured", "Uninjured", "Injured", "Uninjured"))

> text(c(-9,-5), res_post_hoc_injury_rate$k+3, c("Intervention", "Control"))

> par(op)

# Injury risk funnel plot

> funnel(res_injury_rate)

# Generating reports (for reference)

> reporter(res_injury_rate,format="word")

> reporter(res_injury_incidence,format="word")

> reporter(res_post_hoc_injury_rate,format="word")

**Electronic Supplementary Table S1. Full-text Screening Exclusion List**

| Article Name | Author (year) | Reason for Exclusion |
| --- | --- | --- |
| Outcomes of Embedded Athletic Training Services Within United States Air Force Basic Military Training | Fisher et al. (2021) | Wrong intervention |
| Effect of a novel low volume, high intensity concurrent training regimen on recruit fitness and resilience. | Burley et al. (2020) | Wrong intervention |
| Adapted marching distances and physical training decrease recruits' injuries and attrition. | Roos et al. (2015) | Wrong intervention |
| Effects of traditional sit-up training versus core stabilization exercises on short-term musculoskeletal injuries in US Army soldiers: a cluster randomized trial. | Childs et al. (2010) | Wrong comparator |
| Reliability evaluation of functional movement screen for prevention of military training injury: A prospective study in China. | Zeng et al. (2021) | Wrong outcomes |
| Increasing hamstring flexibility decreases lower extremity overuse injuries in military basic trainees. | Hartig & Henderson (1999) | Wrong setting |
| Achilles Tendon and Patellar Tendon Structure in Combat Soldiers Following Prevention Exercises. | Steinberg et al. (2022) | Wrong outcomes |
| Online multifactorial prevention programme has no effect on the number of running-related injuries: a randomised controlled trial. | Fokkema et al. (2019) | Wrong intervention |
| The feasibility of implementing an evidence-based physical training program during a Canadian Armed Forces basic infantry course. | Robitaille et al. (2021) | Wrong setting |
| Predictive Effect of Well-Known Risk Factors and Foot-Core Training in Lower Limb Running-Related Injuries in Recreational Runners: A Secondary Analysis of a Randomized Controlled Trial. | Suda et al. (2022) | Duplicate |
| Prevention of injuries in long-distance runners. | Jakobsen et al. (1994) | Wrong intervention |
| The effects of exercise for the prevention of overuse anterior knee pain: a randomized controlled trial. | Coppack et al. (2011) | Wrong setting |
| Balance and agility training does not always decrease lower limb injury risks: a cluster-randomised controlled trial. | Goodall et al. (2013) | Wrong intervention |
| The Eagle Tactical Athlete Program Reduces Musculoskeletal Injuries in the 101st Airborne Division (Air Assault). | Sell et al. (2016) | Wrong comparator |
| Effectiveness of online tailored advice to prevent running-related injuries and promote preventive behaviour in Dutch trail runners: a pragmatic randomised controlled trial. | Hespanhol et al. (2018) | Wrong intervention |
| Risk of Lower Extremity Injury in a Military Cadet Population After a Supervised Injury-Prevention Program. | Carow et al. (2016) | Wrong setting |
| Effect of static stretching on prevention of injuries for military recruits. | Amako et al. (2003) | Wrong setting |
| A randomized trial of preexercise stretching for prevention of lower-limb injury. | Pope et al. (2000) | Wrong setting |
| A 2-Month Linear Periodized Resistance Exercise Training Improved Musculoskeletal Fitness and Specific Conditioning of Navy Cadets. | Vantarakis et al. (2017) | Wrong setting |
| Gait retraining and incidence of medial tibial stress syndrome in army recruits. | Sharma et al. (2014) | Wrong intervention |
| Influence of the implementation of a comprehensive intervention programme on premature discharge outcomes from military training. | Larsson et al. (2012) | Wrong intervention |
| Injury Prevention Exercises for Reduced Incidence of Injuries in Combat Soldiers. | Steinberg et al. (2021) | Wrong setting |
| Warrior Model for Human Performance and Injury Prevention: Eagle Tactical Athlete Program (ETAP) Part II. | Sell et al. (2010) | Wrong outcomes |
| Effects of a 7-week outdoor circuit training program on Swiss Army recruits. | Hofstetter et al. (2012) | Wrong setting |
| Neuromuscular training with injury prevention counselling to decrease the risk of acute musculoskeletal injury in young men during military service: a population-based, randomised study. | Parkkari et al. (2011) | Wrong intervention |
| Educational online prevention programme (the SPRINT study) has no effect on the number of running-related injuries in recreational runners: a randomised-controlled trial. | Cloosterman et al. (2022) | Wrong intervention |
| Effects of Agility Training on Body Control, Change of Direction Speed and Injury Attrition Rates in Dutch Recruits: A Pilot Study | Dijksma et al. (2019) | Wrong intervention |
| Influence of an injury reduction program on injury and fitness outcomes among soldiers. | Knapik et al. (2004) | Wrong intervention |
| A multiple intervention strategy for reducing femoral neck stress injuries and other serious overuse injuries in U.S. Army Basic Combat Training. | Scott et al. (2012) | Wrong intervention |
| Injury and fitness outcomes during implementation of physical readiness training. | Knapik et al. (2003) | Wrong intervention |
| Risk factors for injuries along an infantry commanders course | Steinberg et al. (2021) | Wrong setting |
| Prevention of overuse injuries by a concurrent exercise program in subjects exposed to an increase in training load: a randomized controlled trial of 1020 army recruits. | Brushoj et al. (2008) | Wrong setting |
| Effects of a pre-training conditioning programme on basic military training attrition rates. | Lee et al. (1997) | Wrong intervention |
| Effectiveness of an 18-week general strength and foam-rolling intervention on running-related injuries in recreational runners. | Desai et al. (2023) | Wrong intervention |
| Core muscle functional strength training for reducing the risk of low-back pain in military recruits: An open-label randomized controlled trial. | Wang et al. (2021) | Wrong setting |
| The effect of a modified physical training program in reducing injury and medical discharge rates in Australian Army recruits. | Rudzki et al. (1999) | Wrong study design |
| Exercise training to prevent anterior knee pain in military recruits. | Divine. (2012) | Wrong study design |
| Core muscle functional strength training for reducing the risk of low back pain in military recruits: An open-label randomized controlled trial. | Wang et al. (2022) | Wrong setting |
| A prospective study on the management of shin splints. | Andrish et al. (1974) | Wrong setting |
| Increasing the physical fitness of low-fit recruits before basic  combat training: An evaluation of fitness, injuries, and  training outcomes | Knapik et al. (2006) | Wrong setting |
| A comparison of sprain and strain injury rates during aerobic/  calisthenic and aerobic/circuit weight training programs | Marcinik et al. (1987) | Wrong setting |
| Effects of ankle dorsiflexion range and pre-exercise calf muscle stretching on injury risk in Army recruits | Pope et al. (1998) | Wrong setting |
| Extreme conditioning programs and injury risk in a US Army Brigade Combat Team | Grier et al. (2013) | Wrong setting |
| Evaluation of a standardized physical training program for basic combat training | Knapik et al. (2005) | Wrong setting |

**Electronic Supplementary Table S2. Eligibility Stage Resolving Conflicts**

| **Author** |  |  |  |  |  | **HW** | **RB** | **Final** |
| --- | --- | --- | --- | --- | --- | --- | --- | --- |
| Slungaard E; Pollock RD; Stevenson AT; Green NDC; Newham DJ; Harridge SDR |  |  |  |  |  | No | Yes | No |
| von Rosen P; Halvarsson B |  |  |  |  |  | No | Yes | No |
| Vincent HK; Brownstein M; Vincent KR |  |  |  |  |  | No | Yes | No |
| Fredericson M; Moore T |  |  |  |  |  | No | Yes | No |
| Baldwin |  |  |  |  |  | No | Yes | No |
| Fredericson |  |  |  |  |  | No | Yes | No |
| Larsen, K.; Weidick, F.; Leboeuf-Yde, C |  |  |  |  |  | No | Yes | No |
| Steinberg N; Funk S; Zeev A; Waddington G; Svorai-Litvak S; Pantanowitz M |  |  |  |  |  | No | Yes | Yes |
| Fokkema T; de Vos RJ; Visser E; Krastman P; IJzerman J; Koes BW; Verhaar JAN; Bierma-Zeinstra SMA; van Middelkoop M |  |  |  |  |  | No | Yes | No |
| Sharma J; Weston M; Batterham AM; Spears IR |  |  |  |  |  | Yes | No | Yes |
| Vantarakis A; Chatzinikolaou A; Avloniti A; Vezos N; Douroudos II; Draganidis D; Jamurtas AZ; Kambas A; Kalligeros S; Fatouros IG |  |  |  |  |  | No | Yes | Yes |
| Chalupa RL; Aberle C; Johnson AE |  |  |  |  |  | Yes | No | No |
| Scott SJ; Feltwell DN; Knapik JJ; Barkley CB; Hauret KG; Bullock SH; Evans RK |  |  |  |  |  | No | Yes | Yes |
| Edouard P; Steffen K; Peuriere M; Gardet P; Navarro L; Blanco D |  |  |  |  |  | No | Yes | Yes |
| Warha, D; Webb, T; Wells, T |  |  |  |  |  | Yes | No | No |
| Shumway JD; Anderson DN; Bishop B |  |  |  |  |  | No | Yes | No |
| Jakobsen BW; Krøner K; Schmidt SA; Kjeldsen A |  |  |  |  |  | Yes | No | Yes |
| Co-Kinetic Journal |  |  |  |  |  | Yes | No | No |
| Roos L; Boesch M; Sefidan S; Frey F; Mäder U; Annen H; Wyss T |  |  |  |  |  | Yes | No | Yes |
| Hofstede, H.; Franke, T.P.C.; van Eijk, R.P.A.; Backx, F.J.G.; Kemler, E.; Huisstede, B.M.A. |  |  |  |  |  | Yes | No | No |
| Knapik JJ; Hauret KG; Arnold S; Canham-Chervak M; Mansfield AJ; Hoedebecke EL; McMillian D |  |  |  |  |  | No | Yes | Yes |
| Zeng J; Zhang RB; Ke JJ; Wu X; Chen LH; Wang YY; Xiao J |  |  |  |  |  | Yes | No | Yes |
| Sell TC; Abt JP; Crawford K; Lovalekar M; Nagai T; Deluzio JB; Smalley BW; McGrail MA; Rowe RS; Cardin S; Lephart SM |  |  |  |  |  | Yes | No | No |
| Parkkari J; Taanila H; Suni J; Mattila VM; Ohrankämmen O; Vuorinen P; Kannus P; Pihlajamäki H |  |  |  |  |  | No | Yes | Yes |
| Grier T; Anderson MK; Depenbrock P; Eiserman R; Nindl BC; Jones BH |  |  |  |  |  | Yes | No | No |
| Sharma, J; Dixon, J; Dalal, S; Heagerty, R; Spears, I |  |  |  |  |  | Yes | No | No |
| Steinberg, N; Zeev, A; Funk, S; Band, SS; Pantanowitz, M; Yavnai, N; Landau, R |  |  |  |  |  | Yes | No | Yes |
| Hazuka, M.L. |  |  |  |  |  | Yes | No | No |
| Larsson H; Tegern M; Harms-Ringdahl K |  |  |  |  |  | Yes | No | Yes |
| Hofstetter MC; Mäder U; Wyss T |  |  |  |  |  | Yes | No | Yes |
| Pihlajamäki H; Parviainen M; Kyröläinen H; Kautiainen H; Kiviranta I |  |  |  |  |  | Yes | No | No |
| Chan ZYS; Zhang JH; Au IPH; An WW; Shum GLK; Ng GYF; Cheung RTH |  |  |  |  |  | Yes | No | No |
| Steinberg, N; Pantanowitz, M; Zeev, A; Band, SS; Funk, S; Nemet, D |  |  |  |  |  | Yes | No | No |
| Martínez-Gramage J; Albiach JP; Moltó IN; Amer-Cuenca JJ; Huesa Moreno V; Segura-Ortí E |  |  |  |  |  | Yes | No | No |
| Fisher, R; Esparza, S; Nye, NS; Gottfredson, R; Pawlak, MT; Cropper, TL; Casey, T; Tchandja, J; de la Motte, SJ; Webber, BJ |  |  |  |  |  | Yes | No | Yes |
| Letafatkar, A; Rabiei, P; Afshari, M |  |  |  |  |  | Yes | No | Yes |

**Electronic Supplementary Table S3. GRADE Evidence Profile**

Grade Evidence Profile

Question: Exercised-based intervention versus running only or placebo exercise for running-related injuries among endurance runners

| Certainty assessment | | | | | | | No. of participants | | Effect | | Certainty | Importance |
| --- | --- | --- | --- | --- | --- | --- | --- | --- | --- | --- | --- | --- |
| No. of studies | Study design | Risk of bias | Inconsistency | Indirectness | Imprecision | Other considerations | Intervention | Control | Relative (95% CI) | Absolute (95% CI) |  |  |
| Injury Risk | | | | | | | | | | | | |
| 7 | RCTs | Serious | Not serious^1^ | Not serious | Not serious^2^ | Not serious | 769 | 759 | **LRR 0.21**  (-0.46, 0.05) | **80 fewer per 1000** (from 173 fewer to 13 more) | 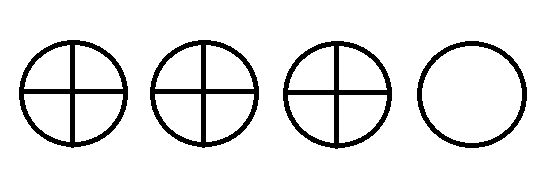  MODERATE | IMPORTANT |
| Injury Rate | | | | | | | | | | | | |
| 6 | RCTs | Not serious | Not serious | Not serious | Not serious | Not serious | 477 | 453 | **LIRR -0.15**  (-0.45, 0.15) | **29 fewer per 10,000h** (from 77 fewer to 19 more) | 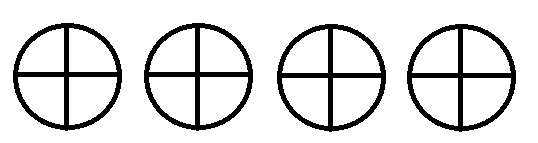  HIGH | IMPORTANT |

**RCT**: Randomized control trials; **CI**: Confidence interval; LRR: Log risk ratio; LIRR: Log incidence rate ratio.

Explanations:

1. Heterogeneity can be explained by the difference between supervised vs. unsupervised studies.
2. There is very little overlap between the confidence interval and RR=1 (LRR=0), thus imprecision is not considered serious.

**Electronic Supplementary Table S4. Summary of Findings**

| **Exercised-based intervention versus running only or placebo exercise for running-related injuries among endurance runners** | | | | | |
| --- | --- | --- | --- | --- | --- |
| **Outcomes** | **Number. of participants (studies)** | **Quality of the evidence (GRADE)** | **Relative effect (95% CI)** | **Assumed risk** | **Absolute effect (95% CI)** |
| **Injury Risk** | 1528 | 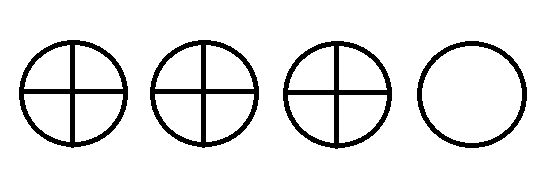  **MODERATE^1^**  due to risk of bias | **LRR 0.21**  (-0.46, 0.05) | **455 per 1000** | **80 fewer per 1000** (from 173 fewer to 13 more) |
| **Injury Rate** | 930 | 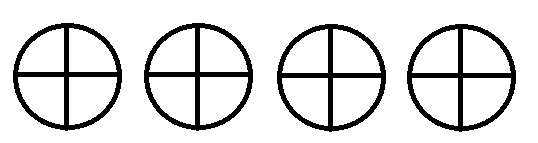  **HIGH** | **LIRR -0.15**  (-0.45, 0.15) | **122 per 10,000h** | **29 fewer per 10,000h** (from 77 fewer to 19 more) |
| **CI**: Confidence interval; **LRR**: Log risk ratio; **LIRR**: Log incidence rate ratio. | | | | | |
| GRADE Working Group grades of evidence  **High Quality**: Further research is very unlikely to change our confidence in the estimate of effect.  **Moderate Quality**: Further research is likely to have an important impact on our confidence in the estimate of effect and may change the estimate.  **Low Quality**: Further research is very likely to have an important impact on our confidence in the estimate of effect and is likely to change the estimate.  **Very Low Quality**: We are very uncertain about the estimate. | | | | | |
| ^1^Risk of bias was deemed to be high for most studies and there is a spread of reasons among deviations from intended interventions, missing outcome, and measurement of the outcome. It is thus decided to downgrade by 1 level. | | | | | |

**Electronic Supplementary Table S5. Meta-analysis Raw Data**

Injury risk meta-analysis

| Trial | author | Citation No. | tpos | tneg | cpos | cneg |
| --- | --- | --- | --- | --- | --- | --- |
| 1 | Baltich et al | [42] | 37 | 49 | 15 | 28 |
| 2 | Bredeweg et al | [43] | 26 | 145 | 32 | 159 |
| 3 | Edouard et al | [44] | 38 | 21 | 50 | 27 |
| 4 | Lundstrum et al | [46] | 13 | 23 | 8 | 3 |
| 5 | Mendez-Rebolledo et al | [47] | 3 | 5 | 7 | 1 |
| 6 | Taddei et al | [48] | 8 | 49 | 20 | 41 |
| 7 | Toresdahl et al | [49] | 188 | 164 | 213 | 155 |

Injury rate meta-analysis

| Trial | author | Citation No. | tcount | texpo | ccount | cexpo |
| --- | --- | --- | --- | --- | --- | --- |
| 1 | Baltich et al | [42] | 40 | 1238 | 16 | 600 |
| 2 | Bredeweg et al | [43] | 26 | 839 | 32 | 1067 |
| 3 | Halvarsson & von Rosen | [45] | 28 | 3214 | 36 | 2979 |
| 4 | Lundstrum et al | [46] | 15 | 1213 | 11 | 577 |
| 5 | Mendez Rebolledo et al | [47] | 4 | 955 | 16 | 906 |
| 6 | van Mechelen et al | [50] | 24 | 4703 | 20 | 4652 |

Post-hoc injury risk meta-analysis

| Trial | author | Citation No. | tpos | tneg | cpos | cneg |
| --- | --- | --- | --- | --- | --- | --- |
| 1 | Lundstrum et al | [46] | 13 | 23 | 8 | 3 |
| 2 | Mendez-Rebolledo et al | [47] | 3 | 5 | 7 | 1 |
| 3 | Taddei et al | [48] | 8 | 49 | 20 | 41 |
